# Smart Markers for Watershed-Based Cell Segmentation

**DOI:** 10.1371/journal.pone.0048664

**Published:** 2012-11-12

**Authors:** Can Fahrettin Koyuncu, Salim Arslan, Irem Durmaz, Rengul Cetin-Atalay, Cigdem Gunduz-Demir

**Affiliations:** 1 Department of Computer Engineering, Bilkent University, Ankara, Turkey; 2 Department of Molecular Biology and Genetics, Bilkent University, Ankara, Turkey; University of Campinas, Brazil

## Abstract

Automated cell imaging systems facilitate fast and reliable analysis of biological events at the cellular level. In these systems, the first step is usually cell segmentation that greatly affects the success of the subsequent system steps. On the other hand, similar to other image segmentation problems, cell segmentation is an ill-posed problem that typically necessitates the use of domain-specific knowledge to obtain successful segmentations even by human subjects. The approaches that can incorporate this knowledge into their segmentation algorithms have potential to greatly improve segmentation results. In this work, we propose a new approach for the effective segmentation of live cells from phase contrast microscopy. This approach introduces a new set of “smart markers” for a marker-controlled watershed algorithm, for which the identification of its markers is critical. The proposed approach relies on using domain-specific knowledge, in the form of visual characteristics of the cells, to define the markers. We evaluate our approach on a total of 1,954 cells. The experimental results demonstrate that this approach, which uses the proposed definition of smart markers, is quite effective in identifying better markers compared to its counterparts. This will, in turn, be effective in improving the segmentation performance of a marker-controlled watershed algorithm.

## Introduction

Automated imaging systems are becoming popular to analyze cellular events of fixed or live cells. These cellular imaging systems have potential not only for decreasing processing time but also for reducing human errors in the analysis. In almost all of the systems, cell segmentation constitutes the first step, which greatly affects the performance of the other system steps. Although there are several algorithms for the segmentation of fixed cell images from a light or a fluorescence microscope, there exist only few for the segmentation of live cells from phase contrast microscopy. In this paper, we focus on the implementation of a robust segmentation algorithm for live cells in culture media.

In general, previous studies have approached the cell segmentation problem in two different contexts: segmenting monolayer isolated cells and segmenting cells that grow in clumps on layers. For monolayer isolated cell segmentation, the studies first differentiate cell pixels from the background using global thresholding [1], adaptive thresholding [2]–[5], and clustering algorithms [6] and then consider the connected components of the cell pixels as the segmented cells.

For the segmentation of clumped cells, the previous studies mainly use active contour models and marker-controlled watershed algorithms. The active contour models define an energy function usually on the edge map of an image, associated with the cell contours, and achieve segmentation by finding the contours that minimize the energy function [7]–[9]. The marker-controlled watershed algorithms identify the markers, each of which corresponds to a cell, and start the flooding process from these markers. One common way to identify the markers is to find regional minima on the intensity/gradient map of the image, reflecting the intensity differences between inside and outside of the cells [10]–[12], and/or on the distance transform of an initially segmented image, reflecting the shape characteristics of the cells [13]–[16]. There are also other methods that are applied on the transforms to find the markers based on the shape characteristics. These methods include applying iterative erosions [17] and modeling by the mixture of Gaussians [18]. As the marker-controlled watersheds typically cause oversegmentation, the studies commonly perform a merge process on the segmented cells after their watershed algorithms [19]–[22].

**Figure 1:**
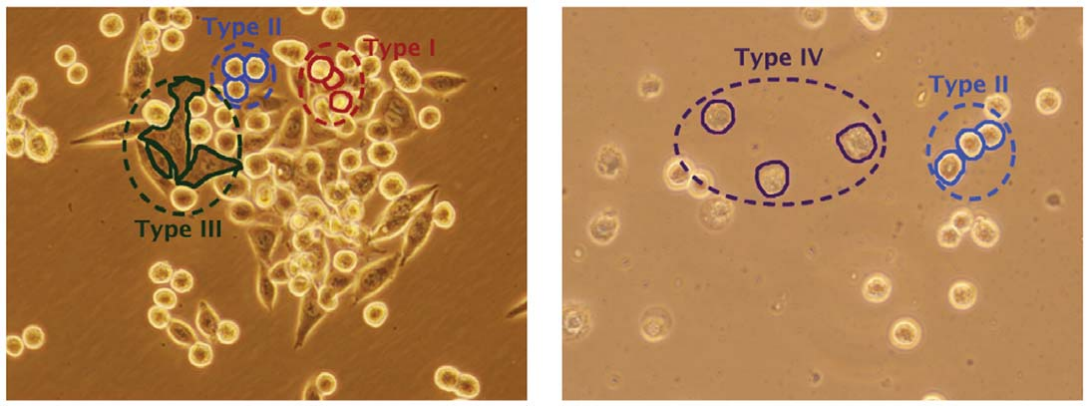
Example images of live KATO-3 gastric carcinoma cells. As shown in the images, these cells can be grouped into four morphological classes based on their visual characteristics. Examples from these groups are also indicated on the images.

Image segmentation in general is an ill-posed problem. The success highly depends on the intent of segmentation as well as the knowledge about the image content. This is especially the case for the problems, in which domain specific knowledge is necessary even for human subjects to achieve successful segmentations. Live cell segmentation is one of such problems. In live cell images, cells of the same cell line or the same tissue may show different morphologies and intensity/texture characteristics. Moreover, these characteristics could be different from a cell line or a tissue to another. For example, KATO-3 gastric cancer cells can be grouped into four morphological classes based on their visual characteristics (Figure 1). The first group corresponds to round cells with relatively brighter inner and boundary pixels. The second one corresponds to round cells as well but these cells consist of relatively darker pixels in their centers and brighter pixels on their boundaries. The third group corresponds to non-circular cells that have relatively larger and irregular shapes and consist of high-gradient dark pixels. These cells also have brighter pixels on their boundaries. The last group corresponds to apoptotic cells whose inner regions and boundaries turn into matte and irregular. The algorithms with the capacity of incorporating this kind of biological knowledge into segmentation have potential to improve the results. This is our main motivation behind using domain specific knowledge, in the form of visual characteristics of the cells, in our segmentation algorithm.

In this paper, we propose a new algorithm for the effective and robust segmentation of live cells. In the proposed algorithm, our main contribution is the incorporation of domain specific knowledge into the definition of a new set of “smart markers” for a watershed algorithm. In order to determine the smart markers, the proposed algorithm identifies different pixel groups with different visual properties, based on the biological background knowledge, and processes these groups with respect to each other, again using the background knowledge of different cell characteristics. Working with live cell images taken from the KATO-3 cell line, our experiments demonstrate that the proposed algorithm, which uses this new smart marker definition, is effective in finding better markers compared to its counterparts, which will in turn improve the segmentation performance of a marker-controlled watershed algorithm. (One should note that the marker term used in this paper is completely different than the one used in immunocytochemistry. Here a marker refers to an image location from which the flooding process of a watershed algorithm starts. The smart marker term is used to indicate that the markers are identified more wisely, considering the visual properties of cells in a cell line.)

The proposed algorithm differs from the previous ones in two main aspects. First, it defines the smart markers based on the background knowledge specific to the image whereas the previous algorithms define them using intensity, gradient, and distance measures without considering the image specific properties. Second, the previous algorithms typically find more markers than the actual cells, resulting in oversegmentation, and hence, they usually necessitate using a merge process after their watershed algorithms. In contrary, the proposed algorithm can find more markers that are one-to-one mapped to the actual cells and can give less oversegmented results without using an external merge process.

## Materials and Methods

### Cell lines

Five different cell lines are used in the experiments. The human gastric cancer cell line (KATO-3) was inoculated in growth medium containing High glucose (4500 mg/L D-Glucose) DMEM with 10% FBS, 1% NEAA, 1% Penicilin/Streptomycin, and 1% L-glutamine. The human liver cancer cell line (Huh7) and the human breast cancer cell line (MCF7) were inoculated in complete growth medium composed of DMEM, with 10% FBS, 1% NEAA and 1% Penicilin/Streptomycin. The human endometrial carcinoma cell line (MFE-296) was cultivated in growth medium containing 40% RPMI 1640, 40% MEM (with Earle salts), 10% FBS, 2 mM L-glutamine and 1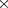 insulin-transferrin-sodium selenite. The human breast cancer cell line (SK-BR-3) was inoculated in complete growth medium composed of HyClone MCCOY'S 5A, together with 10% FBS and 1% L-glutamine. All cell lines were incubated in 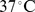, 5% CO2, 95% air containing incubators.

### Smart markers algorithm

**Figure 2:**
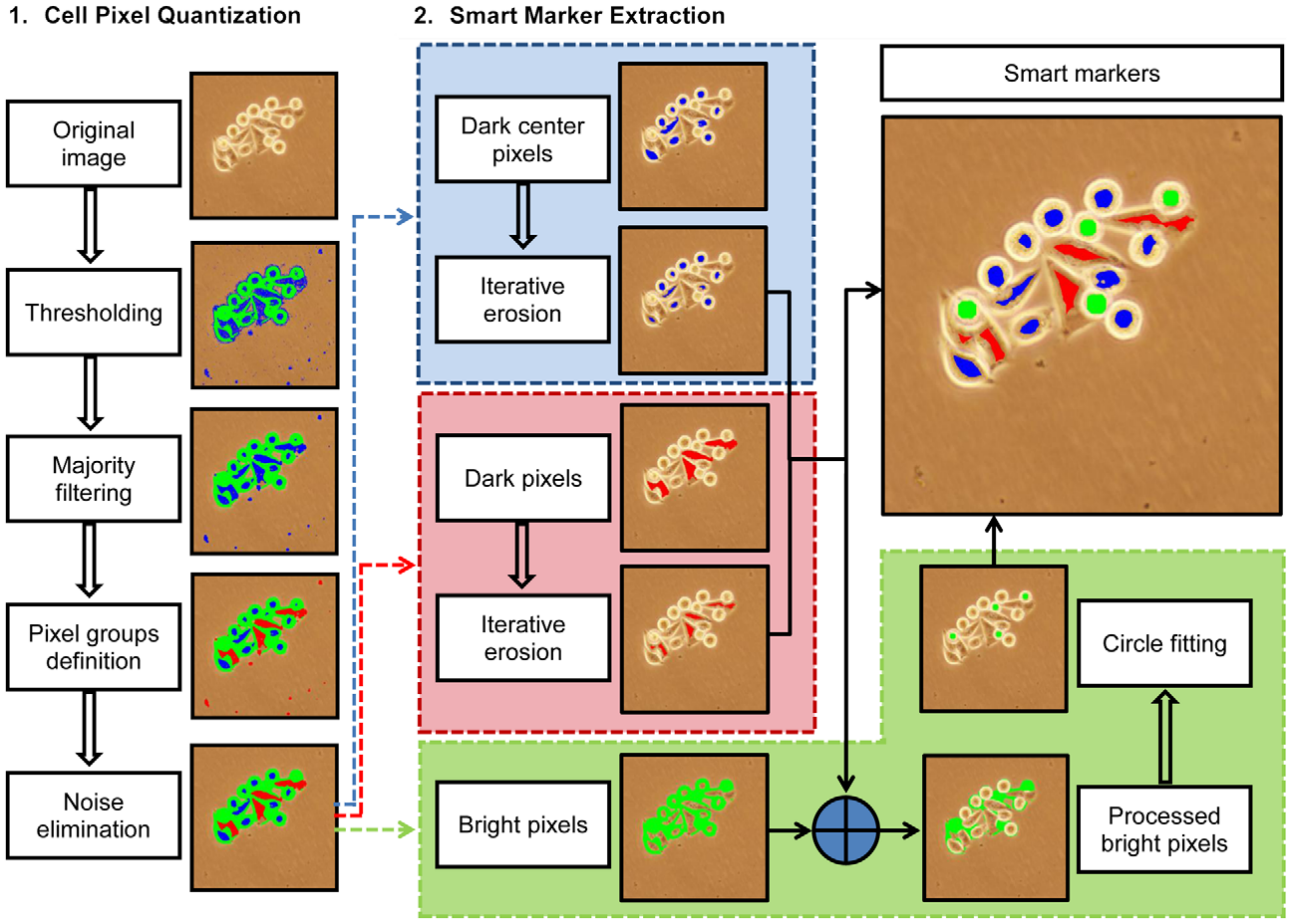
Schematic overview of the proposed algorithm.

The proposed algorithm relies on defining three basic types on image pixels—according to the intensity and gradient of these pixels and their surroundings, associating these basic types with the cells of different characteristics, and extracting the markers on each of these basic types by considering the morphological characteristics of their associated cells. The details of this algorithm are explained in the following subsections. A schematic overview of the algorithm is provided in Figure 2.

In this work, we develop our algorithm focusing on the KATO-3 human gastric cancer cell line. Therefore, we consider the characteristics of its cells in the definition of the basic pixel types and the markers. Nevertheless, this idea can also be applied to other cell lines or tissues, provided that the basic types reflecting the characteristics of their cells are defined. In our experiments, we also obtain preliminary results on four different cell lines to explore the applicability of this algorithm to others.

#### Cell pixel quantization

This part consists of transforming an image into three basic types of pixel groups, each of which corresponds to a cell region of different characteristics. These types correspond to (i) bright pixels, (ii) dark pixels fully surrounded by bright pixels, and (iii) dark pixels only partially surrounded by bright pixels. They are herein referred to as *bright*, *dark-center*, and *dark* pixels, respectively. These three pixel types are used for characterizing the four morphological classes of the KATO-3 gastric cancer cells; these classes are explained in the introduction and illustrated in Figure 1. Particularly, we employ bright pixels for characterizing Type I cells as well as the boundaries of the others, dark-center pixels for Type II cells, and dark pixels for both Type III and Type IV cells.

Cell pixel quantization starts with identifying bright and dark cell pixels. *Bright* pixels correspond to high intensity regions in the image. Hence, we obtain them by thresholding the gray-level image with the Otsu method [23], which automatically computes the threshold 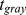 on intensity values. *Dark* pixels correspond to relatively darker regions with high gradient values. Here one should note that dark cell regions have an intensity distribution similar to the background. Thus, using only intensities, without considering gradient values, would yield errors in pixel quantization. In this work, we use the Sobel operators on gray-level intensities to define gradient values. Computing a new Otsu threshold 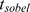 on these gradients, dark pixels are defined as the pixels whose gray-level intensities are less than 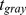 and whose gradients are greater than 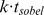. Here the Sobel threshold is multiplied by a constant 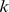 since our experiments reveal that relatively lower gradients should also be considered in the dark pixel definition.

**Figure 3:**
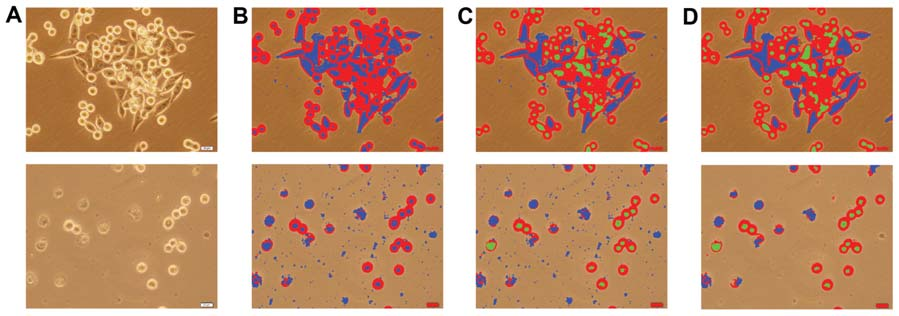
Illustration of the cell pixel quantization step on two exemplary images. (A) Start with an original image, (B) identify *bright* and *dark* pixels using the intensity and gradient information, (C) identify some of dark pixels as *dark-center* pixels, and (D) eliminate noise and artifacts. In this illustration, bright, dark, and dark-center pixels are shown with red, blue, and green, respectively.

After this quantization, dark pixels are further grouped into two based on whether they are fully surrounded by bright pixels; that is, some of the dark pixels are identified as dark-center pixels. However, there usually exists noise in the quantized pixels, which leads to errors in the definition of dark-center pixels. Thus, we postprocess the quantized pixels to alleviate the noise. For that, we first eliminate narrow dark pixel regions around the boundaries of bright regions and then apply a majority filter on the quantized pixels. For the example live cell images shown in Figure 3A, the quantized pixels obtained by this process are illustrated in Figure 3B. In this figure, bright and dark pixels are shown in red and blue, respectively. After this noise elimination, we identify the *dark-center* pixel group as follows: We consider all pixels except the bright ones and find the connected components on these pixels. Let 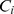 be the i*th* connected component and 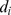 and 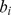 be the numbers of dark and background pixels in the component 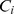, respectively. Dark pixels in 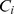 are identified as dark-center pixels if 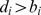. Otherwise, they remain as dark pixels. Figure 3C illustrates the quantized pixels obtained at the end of this step. Here dark-center pixels are shown in green.

The final step is to eliminate holes and artifacts from the pixel groups. First, we fill holes in between cell pixels provided that the holes are smaller than an area threshold 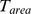. In our experiments, we observe that the main source of noise and artifacts is the dark components. They may correspond to small noisy regions as well as relatively larger artifacts usually found in the background (see the second row of Figure 3). These larger artifacts typically do not contain any bright pixels on their boundaries. Thus, using these observations, we define two rules: First, we eliminate the dark components if they are smaller than the area threshold 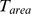. Second, we eliminate the dark components that do not contain any bright pixels on their boundaries. The quantized pixels obtained at the end of these elimination procedures are shown in Figure 3D. We use these quantized pixels to define our smart markers.

#### Smart marker extraction

The proposed algorithm defines the markers for each of the three pixel types separately, according to the characteristics of the regions that each type corresponds to. Since the markers are defined considering the background knowledge of the corresponding region characteristics, it is expected to find more markers that are one-to-one mapped to the actual cells, and thus, to obtain less under and oversegmented results.

**Figure 4:**
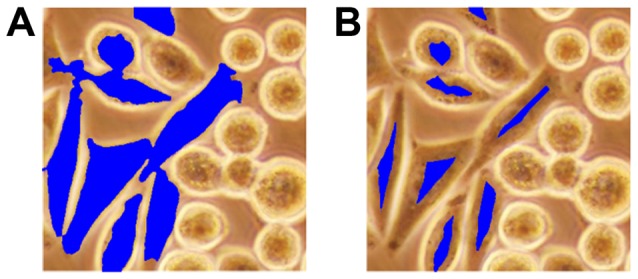
Illustration of the iterative erosion algorithm on *dark* pixels. (A) before and (B) after.

**Figure 5:**
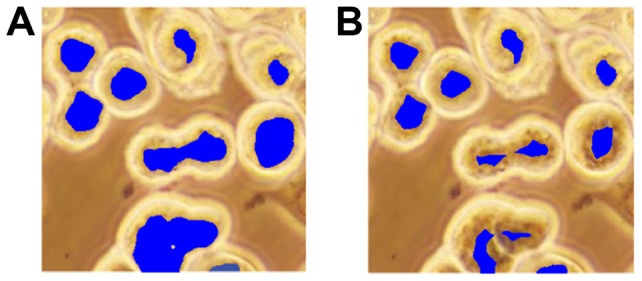
Illustration of the iterative erosion algorithm on *dark-center* pixels. (A) before and (B) after.

In order to define the markers on dark and dark-center pixels, we employ an iterative erosion algorithm. This algorithm erodes the given pixel groups iteratively until the size of a group falls below a threshold. In our work, we select this threshold separately for dark and dark-center pixels, considering their region characteristics. Since dark-center pixels usually correspond to relatively smaller regions compared to dark pixels, we use a size threshold 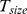 for dark pixels and the half of it (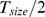) for dark-center pixels. Similarly, we use a disk structuring element with a radius of 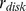 for the erosion of dark pixels and its half (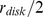) for that of dark-center pixels. The iterative erosion algorithm on dark and dark-center pixels is illustrated in Figures 4 and 5, respectively.

**Figure 6:**
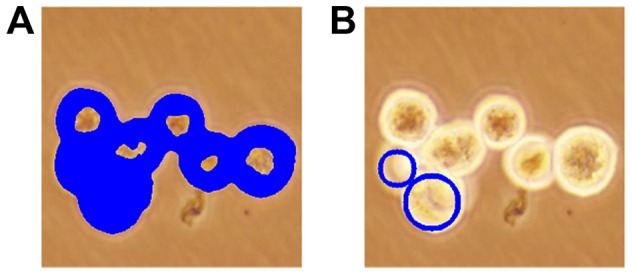
Illustration of the circle-fit algorithm on the *bright* pixels. (A) before and (B) after.

To define the markers on bright pixels, we take the following observation into consideration. Bright pixels can be found both inside a particular class of cells and the boundaries of the others. To alleviate the negative effects of the boundaries, we first dilate the previously found markers and then locate circles on the remaining bright pixels using the modified version of the circle-fit algorithm [24]. In this algorithm, starting from the largest one, we iteratively locate circles on the given pixels provided that the size of a circle is larger than the threshold 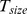 and the circle boundaries are close enough to the non-bright pixels. This circle-fit algorithm on bright pixels is illustrated in Figure 6.

## Results

### Dataset

We conduct our experiments on 44 live cell images of the KATO-3 human gastric cancer cell line. The dataset contains a total of 1954 cells most of which grow in clumps on layers. Each image has a resolution of 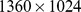 pixels. The images are captured by a digital (Olympus DP72, Tokyo, Japan) microscope with a 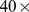 objective lens. The cells are annotated by our biologist collaborators, manually drawing their boundaries. We will use the centroids of these annotated cells for marker-based evaluation and their boundaries for area-based evaluation.

In our experiments, the images are randomly divided into training and test sets. The training set includes 474 cells of 10 different images whereas the test set includes 1480 cells of the remaining 34 images. The cells in the training set are used to estimate the parameters of our algorithm as well as those that we use in our comparisons. The cells in the test set are not used in the parameter estimation at all.

### Evaluation

Marker-controlled watershed algorithms first identify markers on an image and then start the flooding process from these markers. The success of the segmentation is closely related with how well the markers are identified on the image. One can obtain more accurate segmentation results if there is one-to-one correspondence between the markers and the actual cells. Since the correct identification of the markers greatly affects the segmentation results as well as the main contribution of this paper is on the marker definition, in this section, we report the experimental results in terms of the markers, but not the segmentation boundaries. However, it is also possible to apply a watershed algorithm on the markers to obtain the boundaries. This possibility will be explored in the next section.

In our experiments, we evaluate the results both visually and quantitatively. For that, we consider the centroids of the annotated cells as the gold standards and the centroids of the identified markers as the computed cells and use a distance-based evaluation algorithm to obtain the quantitative results. In this marker-based evaluation algorithm, each marker (computed cell) is matched to every gold standard cell provided that the distance between the marker and the gold standard cell is less than a predefined distance threshold. By making use of these matchings, we compute the number of one-to-one matches, oversegmentations, undersegmentations, false detections, and misses, whose definitions are given below. Additionally, we use the *precision*, *recall*, and *F-score* measures in our evaluation.

- A marker (or a gold standard cell) corresponds to *one-to-one match* if the marker is matched to a single gold standard cell that is not matched with any other markers.
- A gold standard cell corresponds to *oversegmentation*, if more than one marker is matched to this gold standard cell. The number of such markers is considered in reporting the quantitative results.
- A marker corresponds to *undersegmentation* if it is matched more than one gold standard cell. The number of such gold standard cells is considered in reporting the quantitative results.
- A marker corresponds to *false detection*, if it is not matched to any gold standard cells.
- A gold standard cell corresponds to *miss*, if none of the markers are matched to this gold standard cell.

### Parameter selection

The proposed algorithm has five external model parameters. The first three of these parameters are used for cell pixel quantization whereas the other two are used for smart marker extraction. These parameters are the Sobel threshold constant 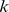, the size 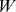 of the majority filter, the area threshold 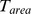, the size threshold 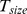, and the radius 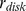 of the structuring element. In our experiments, we consider all possible combinations of the following parameter sets 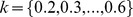, 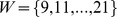, 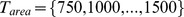, 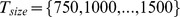, and 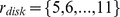. Here we select these parameter sets according to image characteristics. For example, we consider the typical size of a cell and image resolution to determine an initial value for the area threshold 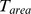 and then include its nearby values to the parameter set.

From all possible combinations of the parameter sets, we select the one that gives the maximum F-score on the training cells. This selection automatically evaluates the combinations based on their F-scores and does not involve any manual or visual examination. After this procedure, the parameters are selected as 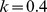, 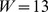, 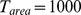, 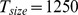, and 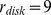.

### Comparisons

We compare our results against those of the three marker identification algorithms. The first is the *intensity-based* algorithm. It defines the markers computing regional minima on gray-level intensities 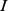 of the given image. Here, to avoid the effects of noise, it uses the 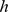-minima transform, which suppresses all minima in the intensity map 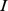 whose depth is less than a scalar 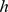.

The second one is the *distance-based* algorithm which is similar to the intensity-based algorithm except that it uses the inverse of the distance transform instead of intensities. It obtains the distance transform map on the initial segmentation of the image such that the minimum distance from each foreground pixel to a background pixel is computed. Similarly, it uses the 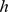-minima transform to reduce the effects of possible noise in the distance map. This algorithm necessitates obtaining an initial segmentation before finding the markers. For that, in our experiments, we use the cell regions that the cell pixel quantization step identifies as the initial segmentation; i.e., the union of bright, dark, and dark-center pixels are used as the initial segmentation. Here we do not use the standard thresholding-based algorithms, which are typically used to obtain initial segmentations, since they yield worse results for our dataset.

The last is the *conditional-erosion* algorithm, which defines the markers on the initial segmentation map of the image by making use of iterative erosions [17]. It first iteratively erodes the connected components of the map with a coarse structuring element while the size of the components is greater than an area threshold. It repeats the same procedure on the resulting components, this time using a fine structuring element and a smaller area threshold. Likewise, we use the union of bright, dark, and dark-center pixels identified by our algorithm as the initial segmentation.

These algorithms also have their own parameters. Besides, the method used to obtain the initial segmentation maps introduces additional ones. In our experiments, we use a similar method to select these parameters: we first list different values for each parameter, consider different combinations of the parameter values, and select the combination that yields the maximum F-score on the training cells.

**Figure 7:**
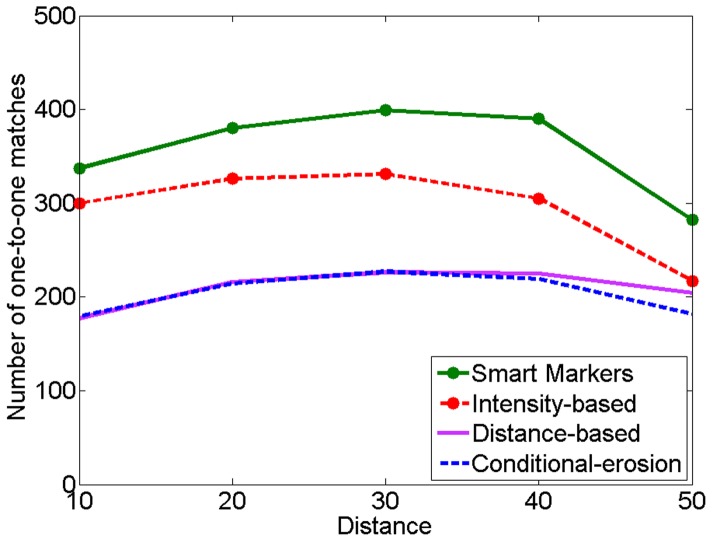
For the training set, the number of one-to-one matches as a function of the distance threshold value used in our marker-based evaluation algorithm.

**Figure 8:**
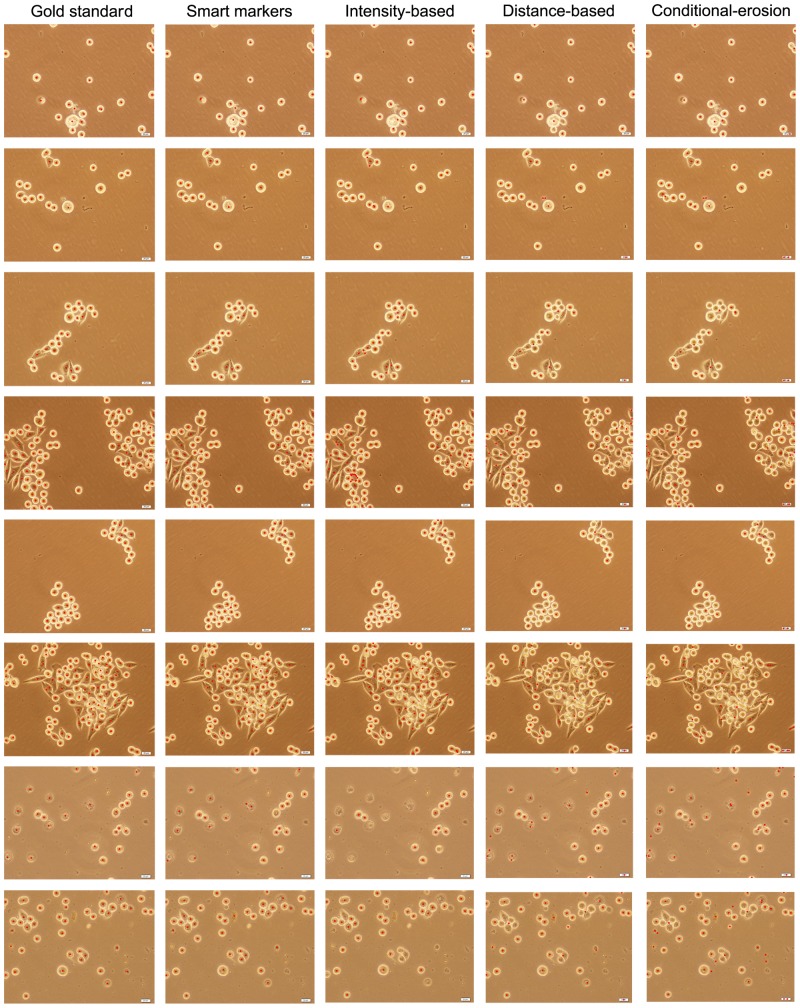
Visual results of the algorithms obtained on example images.

We present the quantitative results obtained on the training and test sets in Tables 1 and 2, respectively. As mentioned before, to obtain these results, we employ a marker-based evaluation method that uses a distance threshold to find matches between the markers and the actual cells. Smaller values of this threshold increases false detections and misses since some of the identified markers are not close enough to the exact centroids of the gold standard cells. This decreases one-to-one matches, giving lower precision and recall values. Its larger values increases oversegmentations since more markers are matched to the same gold standard cell. This also decreases one-to-one matches. Figure 7 shows the number of one-to-one matches as a function of the distance threshold value for the training set. Considering these numbers, we select the distance threshold as 30, which gives the maximum one-to-one matches for all of the algorithms. We also present the visual results obtained for example images in Figure 8.

**Table 1:** Comparison of the proposed smart markers algorithm against different marker identification algorithms.

	One-to-one	Overseg.	Underseg.	False	Miss	Precision	Recall	F-score
Smart markers	408	4	10	29	53	0.92	0.86	0.89
Intensity-based	331	16	14	124	122	0.70	0.70	0.70
Distance-based	245	4	8	92	218	0.71	0.52	0.60
Cond-erosion	231	23	8	97	226	0.65	0.49	0.56

The results are obtained on the training set using marker-based evaluation.

**Table 2:** Comparison of the proposed smart markers algorithm against different marker identification algorithms.

	One-to-one	Overseg.	Underseg.	False	Miss	Precision	Recall	F-score
Smart markers	1284	36	52	122	129	0.88	0.87	0.87
Intensity-based	1102	50	50	138	309	0.84	0.74	0.79
Distance-based	834	17	34	240	604	0.75	0.56	0.64
Cond-erosion	790	109	50	307	601	0.64	0.53	0.58

The results are obtained on the test set using marker-based evaluation.

The results show that the definition of smart markers leads to higher precision and recall values. Compared to the other algorithms, it gives more one-to-one matches with relatively less false detections and misses. In Tables 1 and 2, we observe that the most successful comparison algorithm is the intensity-based algorithm. However, when we examine the visual results (the third column of Figure 8), we observe that this algorithm usually fails in finding Type I cells, which contain bright pixels both in their centers and on their boundaries, and Type IV cells, which correspond to apoptosis. Besides, for images that contain noise and artifacts, it may find a very large number of markers. Indeed, the reported results do not reflect this fact since we mask the markers with the initial segmentation found by our algorithm. If such a masking operation was not used, the number of false detections would increase from 138 to 427.

Moreover, the results show that the distance-based and conditional-erosion algorithms give less one-to-one matches due to a high number of misses. The visual results of these algorithms (the fourth and the fifth columns of Figure 8) reveal that they are not successful in finding clumped cells, regardless of their morphological classes. It is also worth noting that these algorithms require an initial segmentation and the quality of this segmentation greatly affects the final segmentation results. In the experiments, we use the initial segmentation found by our algorithm, which uses domain specific knowledge to define this segmentation. Without using this domain specific knowledge, it may be harder to find a good initial segmentation especially for Type III cells, which correspond to darker and non-circular cells, and Type IV cells, which correspond to apoptosis. This may further decrease the number of one-to-one matches.

### Parameter analysis

**Figure 9:**
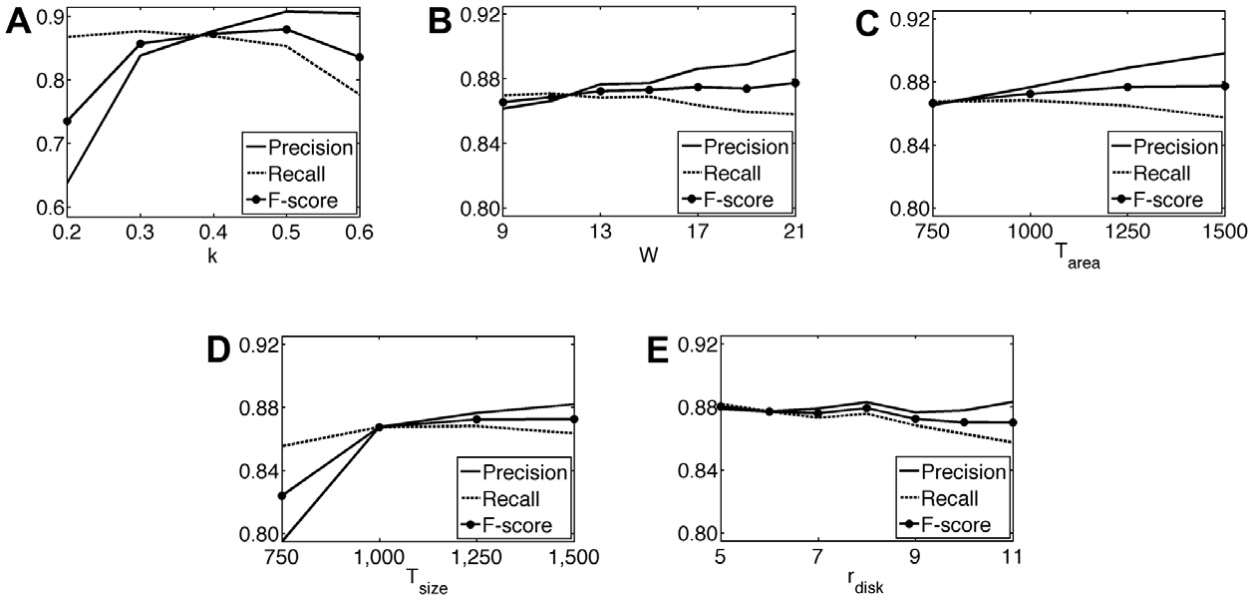
For the test set, the precision, recall, and F-score measures. As a function of (A) the Sobel threshold constant 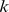, (B) the size 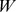 of the majority filter, (C) the area threshold 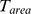, (D) the size threshold 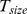, and (E) the radius 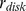 of the structuring element.

The proposed algorithm has five model parameters. To investigate the effects of each parameter to the segmentation performance, we fix four parameters and observe the precision, recall, and F-score measures as a function of the other. In Figure 9, we present the parameter analysis performed on the test set.

There are three external parameters in the cell pixel quantization step. The first one is the Sobel threshold constant 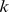 that is used to define dark pixels. When its smaller values are used, some background pixels are also defined as dark so that false background regions are identified as cells. This increases the number of computed cells without increasing one-to-one matches, which in turn lowers precision. On the other hand, when larger values of this constant are used, less dark pixel components can be found. This leads to less computed cells as well as less one-to-one matches, which lowers recall. Note that larger values do not lower precision since the number of computed cells and one-to-one matches decrease concurrently. In our experiments, this parameter is selected as 0.4. Figure 9A shows that this selected value provides a good balance between precision and recall.

The second parameter is the size 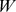 of the majority filter that is used for alleviating the effects of noise in pixel quantization. The filter size 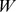 should be selected large enough to get the benefits of majority filtering. On the other hand, selecting too large filter sizes causes to assign incorrect labels to pixels. As seen in Figure 6B, this changes the balance between precision and recall. The area threshold 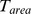 is the last parameter of this step. It is used to eliminate smaller dark components. Smaller threshold values identify more false regions as cells whereas larger values give less computed cells and one-to-one matches. These decrease precision and recall, respectively, as in the case of the parameter 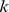. In the experiments, 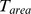 is selected to be 1000, which gives high precision and recall values at the same time (Figure 9C).

There are two parameters used in the smart marker extraction step. These are the size threshold 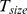 and the radius 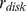 of the structuring element. Smaller values of 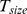 cause to define false markers, increasing the number of computed cells without changing one-to-one matches. On the other hand, its larger values cause to eliminate some true markers, decreasing the number of computed cells as well as one-to-one matches. These two conditions decrease precision and recall values, respectively, as observed in Figure 9D. The radius 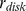 slightly changes the results except the case when largest values are used (Figure 9E). The largest values prevent the iterative erosion algorithm to identify especially smaller true markers; this also lowers recall values. In the experiments, 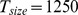 and 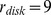, which give a good balance between precision and recall.

## Discussion

In this paper, we introduced the idea of defining smart markers for a marker-controlled watershed algorithm by making use of domain knowledge specific to live cells. This definition relies on defining different pixel groups based on the morphological characteristics of the live cells and identifying the smart markers on these pixel groups. Working with 1954 KATO-3 gastric cancer cells, our experiments indicated the effectiveness of this smart marker definition in obtaining more successful results.

As seen in the visual results (Figure 8), the proposed algorithm can successfully find different types of cells. This is attributed to the fact that the algorithm uses domain specific knowledge so that it knows there exist different types of cells in a cell line (or a tissue) and the characteristics of these cells. Therefore, it can use this knowledge in defining its markers. On the other hand, the other algorithms do not use the knowledge of the existence of different cell types in a cell line. The ability of using such knowledge is indeed closely related with working on live cells. Live cells are not fully attached to the plate, and thus, cells belonging to different morphological classes can show different appearances. On the other hand, when cells are fixed, they become fully attached to the plate and their appearances become the same. The only exception is the appearance of dead (e.g., apoptotic) cells; they usually seem different than the others. Thus, to analyze the morphological classes of fixed cells, special stainings are typically required. The most of the algorithms in literature, including those that we used in our comparisons, were implemented considering fixed cells (mostly for fluorescence stained cells). This could be the reason of these algorithms not considering such kind of knowledge in their segmentations. Our proposed work is a good example of showing how domain knowledge can effectively be used in a cell segmentation algorithm.

**Figure 10:**
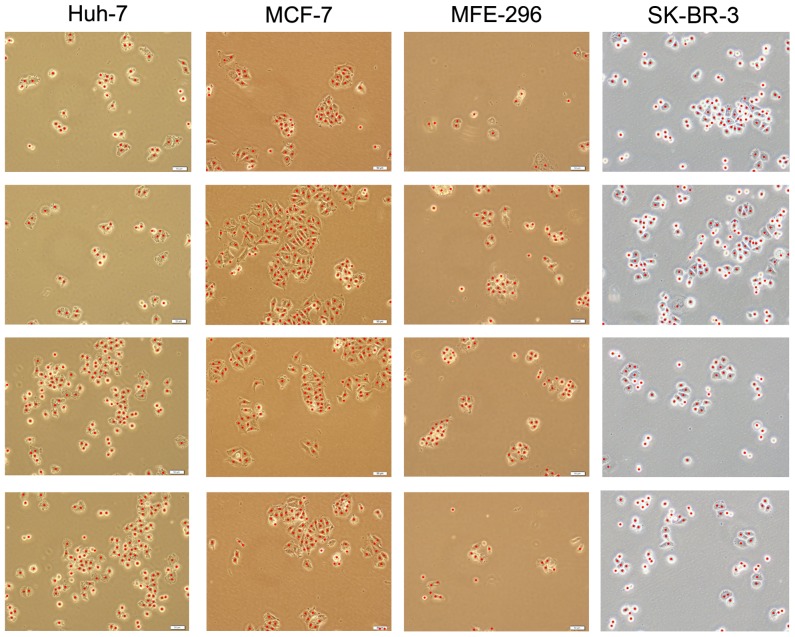
Visual results of the proposed algorithm obtained on the images of different cell lines.

In this work, we developed our algorithm considering the morphological characteristics of the KATO-3 human gastric cancer cell line. We also use the images of this cell line to test our algorithm. Nevertheless, it is also possible to apply this algorithm to other cell lines. To explore this possibility, we also test our algorithm on four different cell lines, namely the Huh7 human liver cancer, MCF7 human breast cancer, MFE-296 human endometrial carcinoma, and SK-BR-3 human breast cancer cell lines. The preliminary visual results obtained on example images of these cell lines are given in Figure 10. This figure shows that the results hold promise for the proposed algorithm to be also used for different cell lines. In order to obtain better results, one can consider the characteristics of these cell lines for the definition of additional pixel groups as well as for the identification of additional smart marker types on these pixel groups. This could be considered as one of the future research directions of our work.

Our experiments showed that the smart marker definition increases the success in terms of marker localization. This, in turn, is expected to also increase the success of a watershed algorithm. To examine this, we implement a watershed algorithm that takes the smart markers as starting locations and grows them by using the marker types and the pixel groups (*dark*, *dark-center*, and *bright* pixels). Here we use the geodesic distance from a pixel to a marker boundary as the growing criterion. Let 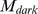, 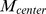, and 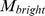 be a set of smart markers defined on dark, dark-center, and bright pixels, respectively. In this watershed, we first grow the markers 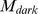 on dark pixels as long as the Euclidean and geodesic distances from a dark pixel to the corresponding marker boundary are equal to each other. This equality constraint is defined to prevent flooding into dark pixels that belong to missing cells with unidentified markers. Then, we repeat the same procedure to grow the markers 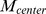 on dark-center pixels. Finally, we combine the grown markers with the centroid of the markers 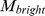 and grow all of them on bright pixels. Here, we identify the most distant pixels that each marker can grow into. For that, for each marker 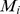, we find the first bright pixel 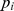 that is adjacent to background and that 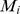 grows into and define the maximum distance as the geodesic distance from 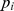 to the closest boundary of 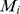 plus an offset value, which is set to 10 in the experiments. This distance constraint is defined to prevent flooding into pixels of missing cells with unidentified markers as well as background pixels that are incorrectly assigned to the bright pixel group. At the end, we postprocess the results by applying the majority filter on the grown areas and filling holes in each segmented cell. For the other algorithms, we grow their markers on their initial masks by considering the same distance constraint and applying the same postprocessing.

**Figure 11:**
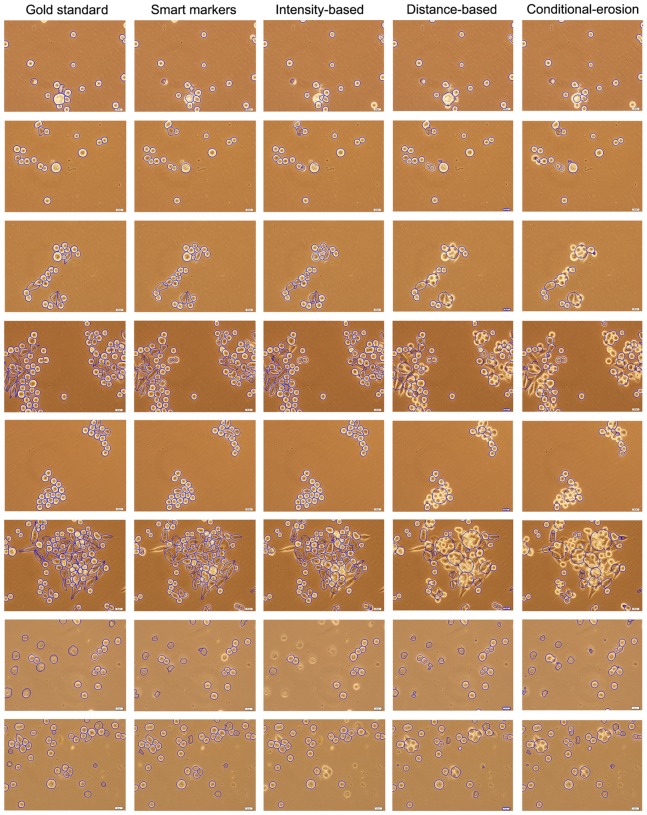
Visual results of the watershed algorithms obtained on example images.

We present area-based evaluation of these watershed algorithms for the training and test sets in Tables 3 and 4, respectively. In this evaluation, we first find the true segmented cells and then calculate the precision, recall, and F-score measures by considering the true positive pixels of these cells. A segmented cell 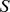 is said to be true if at least half of its pixels overlap a gold standard cell 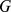 and at least half of the pixels of 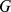 overlap 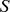. That is, the pixels of a segmented cell are not considered as true positive if there is no one-to-one correspondence between this cell and a gold standard cell. In Tables 3 and 4, we also report the precision, recall, and F-score measures computed on the true segmented cells, without considering their segmented areas. Note that these cell-based results are computed on the segmented cells that are identified as true after the watershed algorithm. Thus, they are less than those computed on the markers before the watershed algorithm. This table reveals that the use of the proposed smart markers gives more successful results than the others in both area-based and cell-based evaluations. We also give the visual comparison on example images in Figure 11. When area-based and cell-based results are assessed together, one can observe that the watershed algorithm that uses the smart markers identifies cells better than finding their exact areas. To improve the segmented areas, one can combine different criteria, such as intensity and gradient values, with the pixel groups in the growing process. This would be another future research direction of this work.

**Table 3:** Comparison of the marker-controlled watersheds that use the smart markers and those identified by the comparison algorithms.

	Area-based			Cell-based		
	Precision	Recall	F-score	Precision	Recall	F-score
Smart markers	0.73	0.67	0.70	0.83	0.77	0.80
Intensity-based	0.71	0.54	0.62	0.75	0.64	0.69
Distance-based	0.50	0.38	0.43	0.64	0.40	0.49
Cond-erosion	0.50	0.35	0.41	0.58	0.38	0.46

The results are obtained on the training set.

**Table 4:** Comparison of the marker-controlled watersheds that use the smart markers and those identified by the comparison algorithms.

	Area-based			Cell-based		
	Precision	Recall	F-score	Precision	Recall	F-score
Smart markers	0.80	0.72	0.76	0.84	0.83	0.84
Intensity-based	0.82	0.66	0.73	0.84	0.74	0.78
Distance-based	0.59	0.47	0.52	0.68	0.50	0.58
Cond-erosion	0.58	0.44	0.50	0.61	0.47	0.53

The results are obtained on the test set.

Our implementation uses C for cell pixel quantization and MATLAB® for smart marker extraction. The average computational time for a single image is 2.63 seconds using a computer with an Intel Core 2 Duo 2.4 GHz processor and 4 GB of RAM. However, it is possible to obtain speedups by implementing the smart marker extraction step also with C. This would be considered as future work.
